# The Cellular and Chemical Biology of Endocytic Trafficking and Intracellular Delivery—The GL–Lect Hypothesis

**DOI:** 10.3390/molecules26113299

**Published:** 2021-05-31

**Authors:** Ludger Johannes

**Affiliations:** Cellular and Chemical Biology Department, Institut Curie, PSL Research University, U1143 INSERM, UMR3666 CNRS, 26 rue d’Ulm, CEDEX 05, 75248 Paris, France; ludger.johannes@curie.fr

**Keywords:** glycosphingolipid, galectin, glycosylation, endocytosis, retrograde trafficking, raft, Casimir force, immunotherapy, tumor targeting, small molecule

## Abstract

Lipid membranes are common to all forms of life. While being stable barriers that delimitate the cell as the fundamental organismal unit, biological membranes are highly dynamic by allowing for lateral diffusion, transbilayer passage via selective channels, and in eukaryotic cells for endocytic uptake through the formation of membrane bound vesicular or tubular carriers. Two of the most abundant fundamental fabrics of membranes—lipids and complex sugars—are produced through elaborate chains of biosynthetic enzymes, which makes it difficult to study them by conventional reverse genetics. This review illustrates how organic synthesis provides access to uncharted areas of membrane glycobiology research and its application to biomedicine. For this Special Issue on Chemical Biology Research in France, focus will be placed on synthetic approaches (i) to study endocytic functions of glycosylated proteins and lipids according to the GlycoLipid–Lectin (GL–Lect) hypothesis, notably that of Shiga toxin; (ii) to mechanistically dissect its endocytosis and intracellular trafficking with small molecule; and (iii) to devise intracellular delivery strategies for immunotherapy and tumor targeting. It will be pointed out how the chemical biologist’s view on lipids, sugars, and proteins synergizes with biophysics and modeling to “look” into the membrane for atomistic scale insights on molecular rearrangements that drive the biogenesis of endocytic carriers in processes of clathrin-independent endocytosis.

## 1. Introduction

The plasma membrane is a selective barrier for controlled exchanges between eukaryotic cells and their environments. Endocytosis describes the cellular process by which parts of the plasma membrane are invaginated to then pinch off membrane-bound carriers for the uptake of material that originates from the extracellular space or the cell surface [1]. Endocytosis thereby serves a multitude of cellular functions that range from nutrient uptake and growth factor signaling to the dynamic maintenance of specialized areas of the plasma membrane, such as the leading edge of migratory cells or the immunological synapse of lymphocytes.

Amongst the endocytic processes that occur in all eukaryotic cells, the clathrin pathway remains by far the best-characterized [2,3,4]. Here, the recruitment of clathrin and its adaptors onto cytosolic signals of transmembrane proteins drives the bending of a patch of plasma membrane into a narrow endocytic pit that then detaches by the action of the scission GTPase dynamin to give rise of an endocytic vesicle.

It is now well established that narrow endocytic pits can also form in endocytic processes that are not driven by the clathrin machinery [5,6,7,8]. A multitude of cellular proteins are involved in these clathrin-independent endocytosis events, such as small GTPases of the Rho/Rac/Cdc42 [9,10] and Arf families [11,12], actin [13,14], galectins [15,16], endophilins [17,18], and reticulon3 [19]. Whether these constitute different endocytic pathways or contribute at different steps to the same endocytic pathway has remained an open question (discussed in Reference [20]). While clathrin-independent endocytic carriers were initially described as pleiomorphic [21], more recent findings identify them in many cases as tubular elements [14,16,17,19,22].

**Figure 1:**
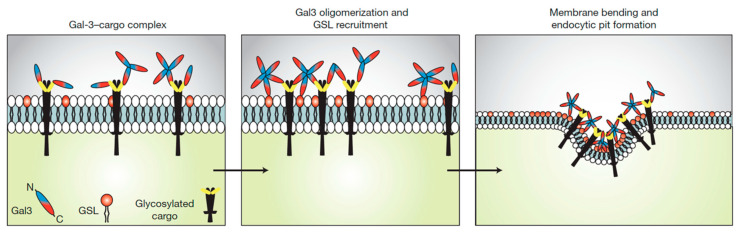
Endocytic pit construction according to the GL–Lect hypothesis. The sugar binding protein galectin-3 (Gal3) is monomeric in solution. It binds to complex carbohydrates of plasma membrane glycoproteins (glycosylated cargo). This leads to Gal3 oligomerization, thereby enabling its capacity to interact with glycosphingolipids (GSLs) in a way such as to induce narrow membrane bending and the clathrin-independent formation of endocytic pits into which the glycosylated cargoes are recruited. From these pit, endocytic carriers are then generated for the cellular uptake of the glycosylated cargoes (not shown). Reproduced with permission from Ref. [16].

In the field of clathrin-independent endocytosis research, the key challenge is to explain how cargo proteins are recruited and the plasma membrane is bent to form endocytic pits without the help of the clathrin coat. It is indeed another common characteristic of clathrin-independent endocytic processes that highly organized electron dense protein assemblies (termed coats) cannot be found at sites of endocytic pit formation. According to the GlycoLipid–Lectin (GL–Lect) hypothesis, oligomeric sugar-binding proteins (lectins) from pathogens or of cellular origin (notably galectins) interact with glycosylated lipids (glycosphingolipids (GSLs) or glycosylphosphatidylinositol (GPI)-anchored proteins) in a way such as to drive narrow membrane bending. This leads to the formation of tubular endocytic pits from which so-called clathrin-independent carriers detach for the cellular uptake of pathogens (e.g., Shiga and cholera toxins, polyomaviruses, norovirus, etc.), or cellular glycoproteins (e.g., integrins, CD44, etc.) that are recruited by galectins [23,24,25,26] (Figure 1). To what extent the GL–Lect hypothesis provides the conceptual framework for a unifying view on clathrin-independent endocytosis remains to be established.

Once internalized, proteins and lipids are sorted to three principal destinations within the endocytic pathway: (i) late endosomes and lysosomes for degradation and nutrient uptake [27]; (ii) back to the plasma membrane for recycling [28]; and (iii) retrograde transport to the Golgi apparatus for subsequent polarized secretion to specialized areas of the plasma membrane, such as the leading edge of migratory cells or the immunological synapse of lymphocytes [29], or further retrograde trafficking to the endoplasmic reticulum (ER) for retro-translocation to the cytosol [30]. For a discussion of the molecular signals that determine sorting on endosomes the reader is referred to excellent recent reviews [31,32,33].

In the context of the GL–Lect hypothesis, many key questions arise on regulation, structural organization, and physiological functions. For this review, in the Special Issue on Chemical Biology Research in France, the focus will be on GL–Lect-related aspects that have been addressed through chemical biology approaches.

## 2. Glycosphingolipids

The mole percentage of glycosphingolipids (GSLs) in biological membranes varies from very low numbers in the nuclear envelop and ER to double digit numbers at the plasma membrane of epithelial cells [34]. GSLs are essential for life from lower organisms such as the fruit fly *D. melanogaster* [35] and the earthworm, *C. elegans* [36], to mammals [37]. They have been ascribed a multitude of functions (e.g., receptor signaling, cell adhesion, membrane trafficking), but molecular mechanisms of action remain poorly explored.

A striking feature of GSLs is their capacity to organize into domains that have been termed lipid rafts [38]. This lateral “connectivity” results from unique features, such as long acyl chains and the capacity to form hydrogen bonds. How raft connectivity is translated into biological functions has mostly remained enigmatic. The raft hypothesis itself has evolved since its initial formulation. While phase separation with raft fabric occurs in passive systems such as liposomes, active cell membranes are understood as non-equilibrium systems. In this context, the construction of raft-type assemblies is thought to be driven by appropriate triggers (e.g., GSL-binding lectins), which thereby transform the “raftophilic” potential of GSLs into defined cell biological outcomes [39,40].

**Figure 2:**
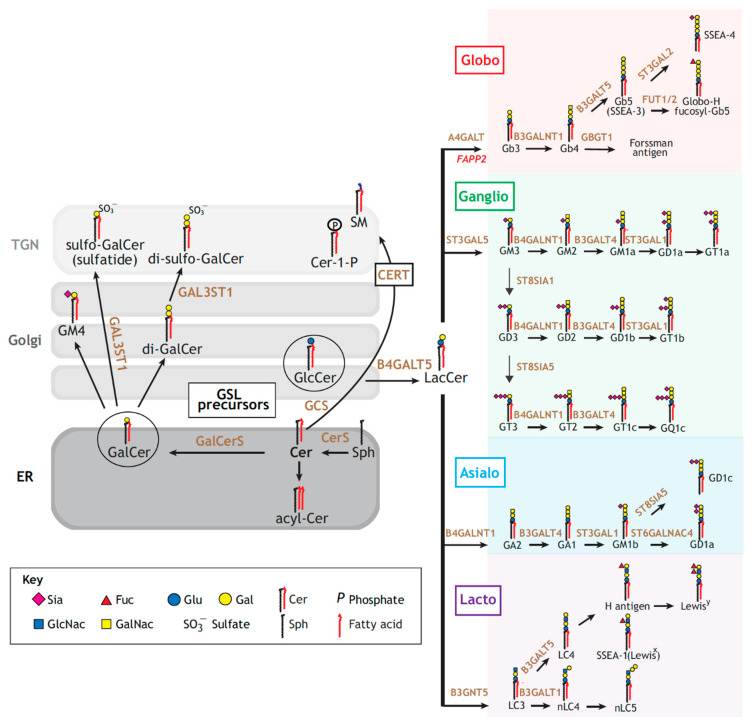
GSL biosynthesis pathways. Left panel: ceramide (Cer) can be acylated (acyl-Cer), phosphorylated (Cer-1-phosphate), or conveyed to the TGN for the synthesis of sphingomyelin (SM). Alternatively, Cer is glycosylated for the synthesis of the GSL precursors, glucosylceramide (GlcCer) or galactosylceramide (GalCer) along the secretory pathway. GalCer is then processed for the production of sulfatides. Right panel: GlcCer is galactosylated to lactosylceramide (LacCer), which serves as a common precursor for the different GSL series: globo (red), ganglio (green), asialo (blue) and lacto (purple). Glycosphingolipid-synthetizing enzymes catalyzing the major synthetic reactions are shown in dark orange. Reproduced with permission from The Journal of Cell Science, Ref. [41].

Hundreds of different GSL species have been described that are made by chains of biosynthesis enzymes [41] (Figure 2). It is thereby virtually impossible to “mutate” the expression of a specific GSL species in the complex cellular environment without affecting the others. Chemical synthesis has provided a means to address this difficulty, which will be exemplified here in the context of the GL–Lect hypothesis.

The bacterial Shiga toxin and the closely related verotoxins from enterohemorrhagic *E. coli* strains are a threat to human health by inhibiting protein biosynthesis in target cells [42,43]. It is the non-toxic homopentameric B-subunit, termed STxB, which drives the clathrin-independent biogenesis of tubular endocytic pits from which the toxins are taken up according to the GL–Lect mechanism (Reference [14], reviewed in Reference [24]). STxB indeed binds to the GSL globotriaosylceramide (Gb3) not only as the cellular toxin receptor, but also to cluster and induce narrow membrane bending. How is this achieved? Synthetic approaches have allowed addressing this question.

**Figure 3:**
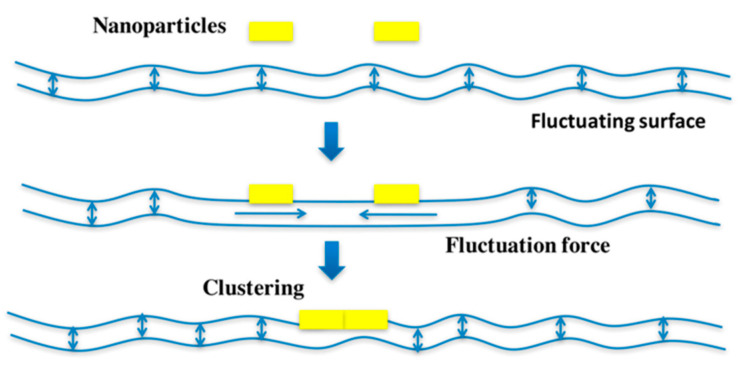
Hypothesis on fluctuation force-driven clustering. Thermally excited fluctuations are suppressed by membrane inclusions (yellow) of nanometric size like STxB, not only at the sites of binding to the membrane, but also for the membrane patch between the two nanoparticles, if they are separated only by a distance of their own size. This perturbation results in an attractive force whose strength is expected to be similar to that of shielded electrostatics or van der Waals interactions. Reproduced with permission from Toxins, Ref. [24].

In solution, STxB homopentamers do not cluster, while they very efficiently do so on membranes. Based on coarse grained dissipative particle dynamics simulations, it was suggested that this occurred through the suppression of thermally excited protrusion fluctuations of membrane lipids between two tightly membrane associated STxB molecules that are at a distance of less than 10 nm from each other [44] (Figure 3). Such fluctuation forces as a source for membrane-mediated clustering had not been suggested before. How should this be tested experimentally? As the first step in this direction, Gb3 molecules were synthesized in which the ceramide part was separated from the globotriose sugar by flexible PEG linkers of increasing length. In dissipative particle dynamics simulations it was shown that with increasing linker length, the suppression of thermally excited protrusion fluctuations became progressively weaker [44]. Under these conditions, clustering was also progressively lost. Satisfyingly, the same trend was observed when STxB clustering was now measured by fluorescence correlation spectroscopy on giant unilamellar vesicles (GUVs) that were made with the corresponding PEG linker types of Gb3 molecules. Furthermore, STxB endocytosis was strongly reduced in cells that were reconstituted with PEG linker Gb3 species, even when the surface density of STxB matched that of cells that were reconstituted with natural Gb3 (Reference [44]). These findings were consistent with the proposal of membrane fluctuation forces as a novel modality to induce heterogeneity in the lateral distribution of GSL-binding proteins in correlated fluids such as biological membranes [45].

The bacterial cholera toxin from *V. cholerae* has a very similar overall structure to Shiga toxin and enters cells by GL–Lect endocytosis via clathrin-independent endocytic carriers [22]. Its B-subunit, CTxB, interacts with the GSL GM1 as the cellular toxin receptor. Using synthetic GM1 species from Reference [46] it was very recently shown that CTxB induced GM1 co-clustering with the GPI-anchored protein CD59 only when the acyl chain of GM1 was fully saturated [47]. Thus, different ceramide species of GM1 dictate their co-assembly with CD59 into CTxB-induced nanodomains, which likely underlies endogenous sorting processes driven by cellular galectins.

**Figure 4:**
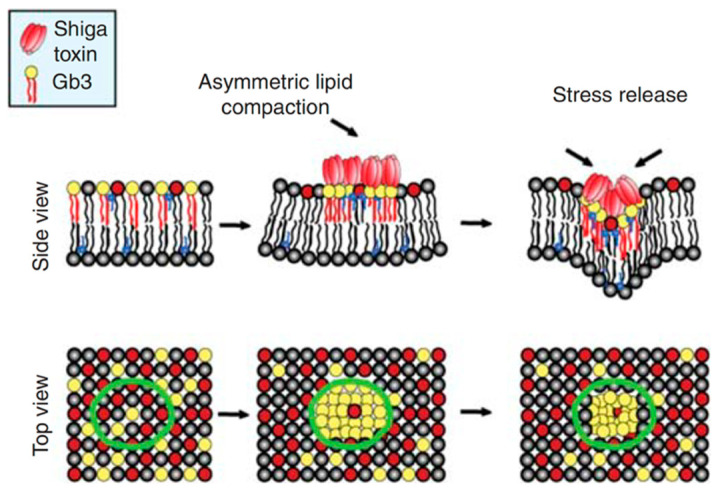
Hypothesis on asymmetric compressive stress leading to negative membrane curvature. Membrane compression of the exoplasmic leaflet to which STxB is bound will force the double-layered plasma membrane to curve in the direction of the toxin. From Reference [20]; courtesy of the Cold Spring Harbor Laboratory Archives.

Synthetic GSL species were also instrumental to propose hypotheses on how the plasma membrane is bent by STxB [14] or CTxB [48] to narrow radii. Using the synthetic PEG linker Gb3 species from Reference [44], it was shown by grazing incidence x-ray diffraction that STxB induced lipid compression in a manner that depended on its mechanical coupling onto Langmuir trough monolayers [49]. This finding was surprising as it is commonly assumed that membranes are difficult to compress. When transposed to bilayered biological membranes, STxB-induced lipid compression would lead to an asymmetric compressive stress and corresponding inward-bound negative curvature (Figure 4), as needed for the construction of uptake sites at the plasma membrane.

**Figure 5:**
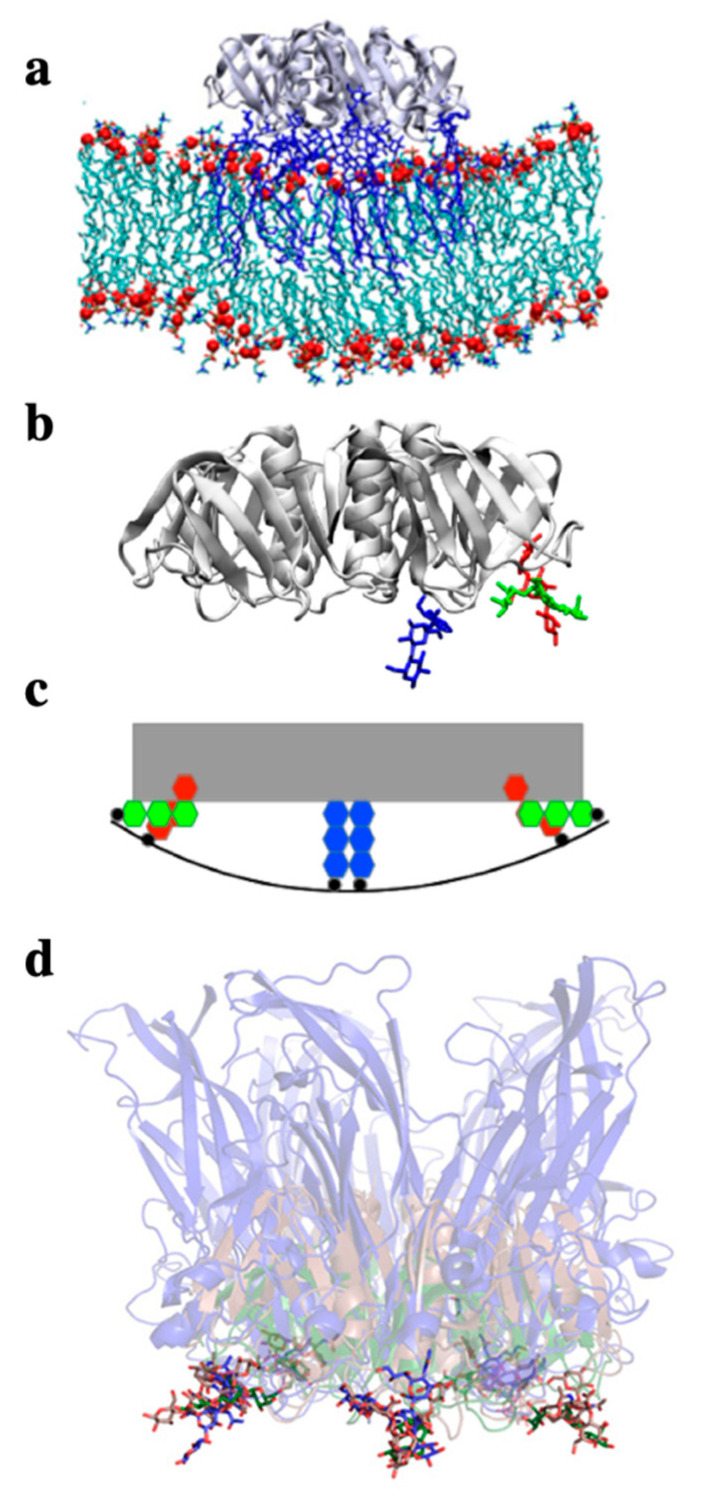
Negative curvature induction based on the geometry of GSL binding sites on lectins. (**a**) All atom molecular dynamics simulation of the STxB homopentamer (gray ribbon structure, side view) on a membrane patch. Gb3 molecules in blue. Note the increment of spontaneous membrane curvature that is observed in silico due to the presence of STxB. (**b**) Representation (extracted from (**a**)) of STxB (side view) with three carbohydrates (blue, green and red). For simplicity, only the three Gb3 binding sites on one monomer are shown. Note that sites 1 (red) and 2 (green) are at the periphery of the protein, while site 3 (blue) faces down under the protein. (**c**) Schematic representation of the situation in (**b**) which illustrates that the membrane (black line) needs to bend up at the edges of STxB to plug the sugars of Gb3 receptor molecules (red and green) into the binding pockets of sites 1 and 2. This would then lead to the generation of negative membrane curvature. (**d**) Overlay of the structures of the following proteins in interaction with GSL receptor analogues: STxB in green with Gb3 (Reference [53]), CTxB in red with GM1 (Reference [54]), the capsid protein VP1 from SV40 in blue (Reference [55]). Note that despite the fact that these proteins do not have any sequence similarity, they fold such that the conserved GSL binding site 2 is presented with the same geometry in space (the sugars of the three structures overlay). It therefore seems likely that this fold was selected by convergent evolution for the same function: to generate negative membrane curvature for building endocytic sites for clathrin-independent endocytosis. Reproduced with permission from Toxins, Ref. [24].

This asymmetric compressive stress mechanism would be expected to synergize with negative curvature induction that results from the specific geometry of GSL binding sites on STxB [50] and CTxB [51,52]. It has indeed been noticed that proteins that do not have any sequence similarity (i.e., STxB and CTxB, but also the VP1 capsid proteins of simian virus 40) fold, such as to present binding pockets for the sugars of their GSL receptors in the same position and orientation at the periphery of the pentameric “doughnut”-like shapes that they adopt (reviewed in references [6,23,24]) (Figure 5). Of note, these binding pockets are located at a distance above the normal plan of the membrane, such that the membrane must bend up at the edges to allow the GSL sugars to reach these sites. It remains to be seen whether this type of molecular organization is also found for the oligomeric galectins.

**Figure 6:**
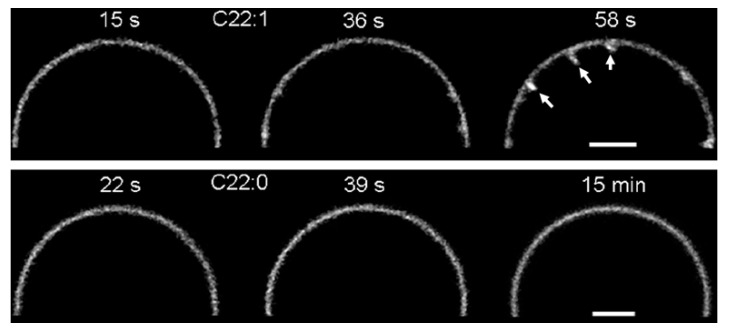
Induction of tubular membrane invaginations by fluorescently labeled STxB on Gb3-containing GUVs. Note that less than 1 min after addition of STxB, tubular membrane invaginations (arrows) were observed on GUVs that contained a C22 Gb3 species with a single unsaturated acyl chain (top panel, C22:1)). In contrast, no tubules were observed on GUVs that contained a C22 Gb3 species with fully saturated acyl chain (bottom panel, C22:0), even after dozens of minutes of incubation with STxB. Bars = 5 µm. Reproduced with permission from Ref. [14].

The use of synthetic Gb3 species allowed to show the capacity of STxB to induce tubular membrane invaginations on GUVs only in the presence of single unsaturated acyl chains, and not fully saturated chains [14] (Figure 6). Based on all atom molecular dynamics simulations it was suggested that this was not because STxB lost its capacity to bend membranes as such [50]. Rather, the raft-type membrane patch that was assembled by STxB became too rigid to be bent when it only contained fully saturated Gb3 species [50]. Of note, for the highly related cholera toxin, endocytic and retrograde trafficking into cells was much more efficient with single unsaturated GM1 acyl chain species, when compared to the fully saturated ones [56]. This finding indicated that the capacity to organize a bending-compatible raft-type nanodomain was indeed critical for the biological effect of the toxins.

## 3. Small Molecule Inhibitors to Study Shiga Toxin Trafficking

One of the best-known facets of chemical biology is the use of small molecule bioprobes to perturb cellular processes and to determine the consequences, which may then lead to the discovery not only of novel molecular mechanisms, but also of more efficient therapeutic drug candidates [57,58,59,60,61]. Due to the possibility to select for survival, toxins have been the target for small molecule-based intervention strategies [62], including the prototypical GL–Lect cargo protein Shiga toxin [63].

In one study, a small molecule screen on cells has led to the identification of two compounds, termed Retro-1 and Retro-2, which slowed the intracellular progression of Shiga toxin towards the ribosomes, its cellular target in the cytosol, specifically at the retrograde trafficking step between early endosomes and the Golgi apparatus [64]. Of note, Retro-2 was the first small molecule which had a protective effect in animal models against Shiga toxin [65] or the plant toxin ricin [64]. An optimized version of Retro-2, termed Retro-2.1, was obtained through a medicinal chemistry approach [66,67,68]. It was then shown that Retro-2.1 protected against a number of other pathogens and pathogenic factors, including several viruses, Leishmania and Chlamydiales [69]. 

**Figure 7:**
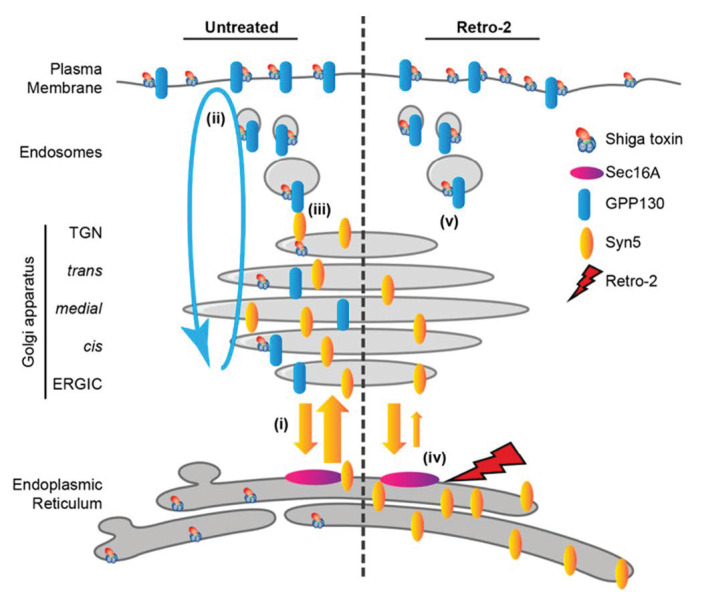
Model of how Retro-2 induces a block of Shiga toxin trafficking at the level of early endosomes. In untreated conditions (left), Shiga toxin trafficking from early endosomes to the TGN requires the interaction of GPP130 (blue) with syntaxin-5 (Syn5, yellow). From the TGN, Shiga toxin then moves on to the ER from where the catalytic A-subunit is translocated to the cytosol to inactivate ribosomes. Under Retro-2 treatment conditions (right), the anterograde transport of syntaxin-5 between ER and Golgi is slowed down by the interaction of Retro-2 with Sec16A, leading to the partial depletion of syntaxin-5 in the Golgi, a loss of its interaction with GPP130, and thereby the blockage of STxB in early endosomes. Reproduced with permission from Ref. [70].

In the initial study, it was found that Retro-1 and Retro-2 induced the redistribution of the trafficking factor syntaxin-5 from the Golgi apparatus to the ER [64]. How this was related to the accumulation of Shiga toxin in early endosomes under Retro compound treatment conditions had remained unexplained. In a recent study, a model for this has been proposed based on the identification of a Retro-2 interacting partner, the ER exit site protein Sec16A [70] (Figure 7). In this study, a clickable version of Retro-2 was synthesized, and Retro-2 interacting proteins were pulled down and identified by mass spectrometry. Sec16A was one of these. Through a yet-to-be-determined mechanism, the binding of Retro-2 to Sec16A was found to slow down the anterograde transport of COPII cargo syntaxin-5 from the ER to the Golgi apparatus. This then also led to the loss of the interaction of syntaxin-5 with another protein, termed GPP130, which was previously shown to be important for Shiga toxin trafficking from early endosomes to the Golgi apparatus [71]. Indeed, a mutant of GPP130 that failed to interact with syntaxin-5 also failed to enable the retrograde trafficking of Shiga toxin to the Golgi. With this model, it could be explained how an effect of Retro-2 on a cellular target in the ER could cause Shiga toxin accumulation in early endosomes (Figure 7). Using small molecules as tools, this study has provided novel cell biological insights on protein sorting at ER exit sites and at the level of the trans-Golgi network. It also helped to further qualify Retro-2 as a broad-spectrum drug candidate for several pathogenic viruses, bacteria, and toxins.

## 4. Vectorial Proteomics

Retrograde trafficking between early endosomes and the Golgi apparatus was discovered using the GL–Lect cargo protein Shiga toxin [72]. This trafficking interface has since become a hot spot of membrane biology research [73,74,75]. While it was initially thought that very few cargo proteins would undergo retrograde transport from the plasma membrane to the Golgi [76], this had not been addressed in a systematic manner. For this, a vectorial proteomics approach was designed [77,78,79]. To generate a capture reagent in the Golgi, the SNAP tag [80] was fused to the transmembrane region of Golgi-localized galactosyl transferase, which itself was fused to GFP for pull-down using GFP-trap beads. The SNAP tag reacts specifically with the small molecule compound O^6^-benzylguanine (BG), which is a synthetic derivative of guanine. It was thereby possible to covalently capture retrograde cargo proteins in the Golgi provided that these had first been labeled at the plasma membrane with BG. For this, a BG labeling reagent was needed with a key property: to be membrane impermeable. This was achieved by synthesizing a BG-containing molecule that had an N-hydroxysuccinimide (NHS) moiety for the reaction with primary amines of plasma membrane proteins, and a PEG9 moiety to provide membrane impermeability.

The first retrograde proteomics study with this system revealed that an unexpectedly large number of plasma membrane proteins underwent retrograde transport to the Golgi [81]. Proteomics results of course need to be confirmed. For this, the cell adhesion and migration factor α5β1 integrin was chosen for which retrograde trafficking had never been described before. In experiments on cells in culture, in *C. elegans* and in mice, it was shown that retrograde trafficking of specifically the non-ligand-bound inactive conformation of α5β1 integrin allowed the protein to have access to the polarized secretion capacity of the Golgi. In migratory cells, retrograde transport was thereby required for the dynamic localization of α5β1 integrin to the leading edge toward which the Golgi is polarized in these cells.

Of note, the conceptual pattern of “retrograde transport to the Golgi to enable polarized secretion to a specialized area of the plasma membrane for the dynamic localization of proteins thereto” may apply more widely [29]. For example, using the Golgi SNAP tag/NHS-PEG9-BG system it was also discovered that in lymphocytes the signaling protein Lat underwent retrograde trafficking and subsequent polarized secretion to be dynamically localized to a specialized plasma membrane area of T lymphocytes: the immunological synapse [82].

The SNAP tag approach has also been exploited to quantify the passage of biological or therapeutic macromolecules from the outside of cells to the cytosol [83]. Macromolecules indeed do not readily cross membranes. Cytosolic arrival, via direct translocation across the plasma membrane or endocytosis followed by endosomal escape, is currently one of the main bottlenecks for the development of macromolecular therapeutics such as nucleic acids (siRNAs, mRNAs, etc.) or peptides [84,85,86]. To quantify the absolute amounts of macromolecules that reach the cytosol, the SNAP tag-based capture reagent, here fused to neon green fluorescent protein, was expressed in the cytosol of target cells. Organic synthesis was used to build chemical branches with (i) BG for reaction with the cytosolic SNAP tag; (ii) NHS for coupling to the macromolecules; and (iii) biotin for ELISA-based quantification [83]. With this setup, it has become possible not only to compare translocation efficacy in different experimental conditions, but also to determine absolute amounts of macromolecules that reach the cytosol. For STxB, this number turned out to be 23,000 homopentamers per HeLa cell after 4 hours of incubation at 37 °C with 40 nM of the vector. For a total of 4.9 million STxB homopentamers that were associated on average with each HeLa cell at this time point, 0.46% had thereby reached the cytosol in a reaction that was sensitive to temperature, cellular ATP levels, and endosomal acidification [83]. This quantitative assay may now allow to further develop STxB as a therapeutic delivery tool (see below) by increasing its intrinsic capacity to escape from endosomes to the cytosol [87].

## 5. Therapeutic Delivery

Antibodies have become powerful macromolecular therapeutics against a wide range of diseases, including notably cancer [88]. Several of them are amongst the best-selling biologicals [89]. Two developments have created an additional momentum: The emergence to the market of antibody-drug conjugates for direct tumor targeting [90], and of immune checkpoint inhibitors for antitumor immunotherapy [91,92].

In a recent prioritization of cancer antigens by the National Cancer Institute at Rockville (USA), 4 out of 75 were GSLs (GD2, GD3, fucosyl-GM1, and GM3), with GD2 arriving already at the 12th position [93]. Yet, GSLs are only rarely exploited for tumor targeting. Generating high affinity binding ligands against GSLs is a notoriously difficult task due to the high structural flexibility of these molecules. An exception is the anti-GD2 antibody dinutuximab, which entered the market for the treatment of high-risk neuroblastoma patients [94].

The GL–Lect cargo protein STxB, a natural GSL ligand, was therefore developed as an alternative scaffold for antibodies to exploit GSLs as therapeutic targets. In an initial approach, it was attempted to target the natural STxB receptor, Gb3, which is overexpressed by certain tumors (e.g., breast cancer [95], colorectal carcinoma [96,97,98], gastric adenocarcinomas [99], pancreatic cancer [100], and lymphomas [101]; reviewed in Reference [102]). In animal models it was shown that STxB accumulated on Gb3 positive tumors in vivo [97,103,104], and that the poorly immunogenic protein could target these tumors even after repeated injections [97]. Synthetic chemistry approaches were used to generate autoimmolative linker arm-based conjugates between STxB and the following cytotoxic molecules: the topoisomerase I inhibitor SN38 (Reference [105]), the pro-apoptotic benzodiazepine RO5-4864 (Reference [106]), the intercalating drug doxorubicin and the highly cytotoxic tubulin polymerization inhibitor monomethyl auristatin F (MMAF) [107,108]. On cells in culture, these conjugates had excellent specificity for Gb3 expressing tumors, and for STxB-MMAF activities in the low nanomolar range were measured [107,108]. In mouse tumor models, these conjugates were unfortunately not successful, most likely due the high Gb3 expression levels on kidney. Very recently, a proprietary methodology has been designed to select for STxB variants that recognize GSLs of choice (patent filing WO2018192719A1). It might thereby become possible to evolve STxB into an alternative molecular scaffold for the targeting of truly tumor specific GSLs or cocktails thereof.

The therapeutic success of inhibitory antibodies against the immune checkpoint molecules PD-1/PD-L1 or CTLA-4 has boosted the general interest in antitumor immunotherapy. Yet, only a fraction of patients (below 30%) respond to checkpoint inhibitory antibodies, and those who respond have tumors that are infiltrated by anti-tumor T cells [109]. It is therefore assumed that vaccines that induce such anti-tumor T cells would synergize with checkpoint inhibitor treatments.

The GL–Lect cargo protein STxB is one vaccine candidate. Its cellular receptor, the GSL Gb3, is expressed on human and mouse dendritic cells [110,111,112]. When chemically coupled to antigenic peptides or proteins, STxB was shown to have a protective effect in mouse models of viral infection [113] or tumor development [114,115,116,117,118,119,120,121,122].

Several aspects of STxB as a vector for immunotherapy are of particular interest. First, it was shown early on that STxB synergized with antibodies against the immune checkpoint molecule PD-L1 in the protection of mice against head-and-neck cancers [117]. Second, STxB coupled to the epitopes of the E7 protein from human papilloma virus 16 induced humoral IgA and cellular CD8+ immune responses in the mucosa of the respiratory tract specifically only when given via the mucosal route of vaccination [116,117,118,119]. Third, when used as a mucosal vaccine, STxB-E7 induced resident memory CD8+ T cells, which are thought to be the most effective cells for controlling tumor growth [121,122]. It has also been argued that only the intranasal route of immunization would lead to sterile immunity against SARS CoV-2 (Reference [123]), and that cellular and humoral immunity are required for optimal protection against the virus [124]. Forth, STxB has recently been obtained by chemical synthesis and in vitro refolding of the protein (patent filing WO/2020/245321). This provides new opportunities for the chemical development of the vector and its industrial production.

## 6. Conclusions

For this Special Issue on Chemical Biology Research in France, the contribution of organic chemical synthesis to the fundamental and applied research exploration of the GL–Lect hypothesis was illustrated. Due to its universal nature, this hypothesis has the potential to become a paradigm in the field of membrane biology, complementary to the clathrin coat paradigm. With the chemical synthesis of STxB and the possibility to create novel GSL binding specificities, unprecedented opportunities furthermore arise for the development of this platform technology towards the clinics: (i) for immunotherapeutics with optimal cross-priming potential against tumor and infectious disease antigens, including SARS CoV-2; and (ii) for tumor targeting tools against GSLs as groundbreaking opportunities in the antibody-drug conjugate market, which, despite its dynamics and economic importance, remains in need for further technological leverage.
